# Regulating IRFs in IFN Driven Disease

**DOI:** 10.3389/fimmu.2019.00325

**Published:** 2019-03-29

**Authors:** Caroline A. Jefferies

**Affiliations:** Department of Medicine, Division of Rheumatology and Department of Biomedical Sciences, Cedars Sinai Medical Center, Los Angeles, CA, United States

**Keywords:** interferon, ubiquitin, E3 ligase, microRNA, monocyte

## Abstract

The Interferon regulatory factors (IRFs) are a family of transcription factors that play pivotal roles in many aspects of the immune response, including immune cell development and differentiation and regulating responses to pathogens. Three family members, IRF3, IRF5, and IRF7, are critical to production of type I interferons downstream of pathogen recognition receptors that detect viral RNA and DNA. A fourth family member, IRF9, regulates interferon-driven gene expression. In addition, IRF4, IRF8, and IRF5 regulate myeloid cell development and phenotype, thus playing important roles in regulating inflammatory responses. Thus, understanding how their levels and activity is regulated is of critical importance given that perturbations in either can result in dysregulated immune responses and potential autoimmune disease. This review will focus the role of IRF family members in regulating type I IFN production and responses and myeloid cell development or differentiation, with particular emphasis on how regulation of their levels and activity by ubiquitination and microRNAs may impact autoimmune disease.

Interferon regulatory factors (IRFs) are a family of transcription factors that regulate many aspects of innate and adaptive immune responses—including driving anti-viral responses, responding to pathogens to drive pro-inflammatory responses and regulating immune cell differentiation (1). Comprised of 9 family members, the IRFs share significant homology within their N-terminal DNA-binding domain (DBD) of ~120 amino acids which forms a helix-loop-helix motif that recognizes specific DNA sequences similar to the interferon stimulated response element (ISRE). The C terminal domain is more diverse amongst family members and confers their unique function via regulating their ability to interact with each other and proteins outside of the IRF family. In general, the C terminal domain of each IRF member contains a nuclear export sequence, an autoinhibitory sequence, and an IRF-association domain which for most family members contains serine residues that are phosphorylated to regulate activity. IRF family members can both homodimerize and heterodimerize, forming both transcriptionally active or repressive complexes as discussed below [reviewed extensively elsewhere (1–3)]. Given their central role as transcriptional regulators of type I Interferon (IFN-α and -β) biology, they have been implicated in in the pathology of several autoimmune and autoinflammatory conditions, including systemic lupus erythematosus (SLE) in which overexpression of type I IFNs is thought to be a major contributor to pathology (4, 5).

This review will address the role of IRF family members in regulating type I IFN production and responses and myeloid cell development or differentiation. Specifically, it will focus on providing an update on how regulation of their levels and activity by microRNAs or ubiquitination may impact IFN-driven autoimmune disease.

## IRF Family—Role in Type I IFN Biology

The type I IFN system comprises 13 subtypes of IFN-α, in addition to IFN-β, IFN-ε, IFN-λ, and IFN-θ (6, 7). The main function of these cytokines is to direct anti-viral immunity: promoting differentiation of B cells into antibody producing plasma cells, inducing differentiation of naïve T cells to effector CD4 or CD8 T cells, reducing proliferation of Treg cells and driving the expression of MHC class I and II and costimulatory molecules on dendritic cells and monocytes (8). Under normal homeostatic conditions, IFN-α and IFN-β are produced in response to detection of viral RNA and DNA by pattern recognition receptors (PRRs). Toll like receptors 3, 7, and 9 are the canonical and best described of the PRRs that recognize viral RNA and DNA, but in more recent years cytosolic PRRs that detect intracellular RNA and DNA, such as RIG-I, c-GAS, and DDX41 have been recognized as key drivers of the antiviral response and type I IFN production [reviewed in (9)]. Both TLRs and cytosolic RNA and DNA can also recognize self RNA/DNA and drive the production of type I IFNs also. Self RNA and DNA released from dead or dying cells is detected by the endosomal TLRs, TLR3, 7, and 9, whilst damaged DNA or oxidized DNA released from damaged mitochondria is detected by cytosolic DNA sensors (10). These pathways are the primary drivers of IFN overproduction and IFN-driven pathology in SLE (11).

### IRFs as Regulators of IFN Expression

**Figure 1:**
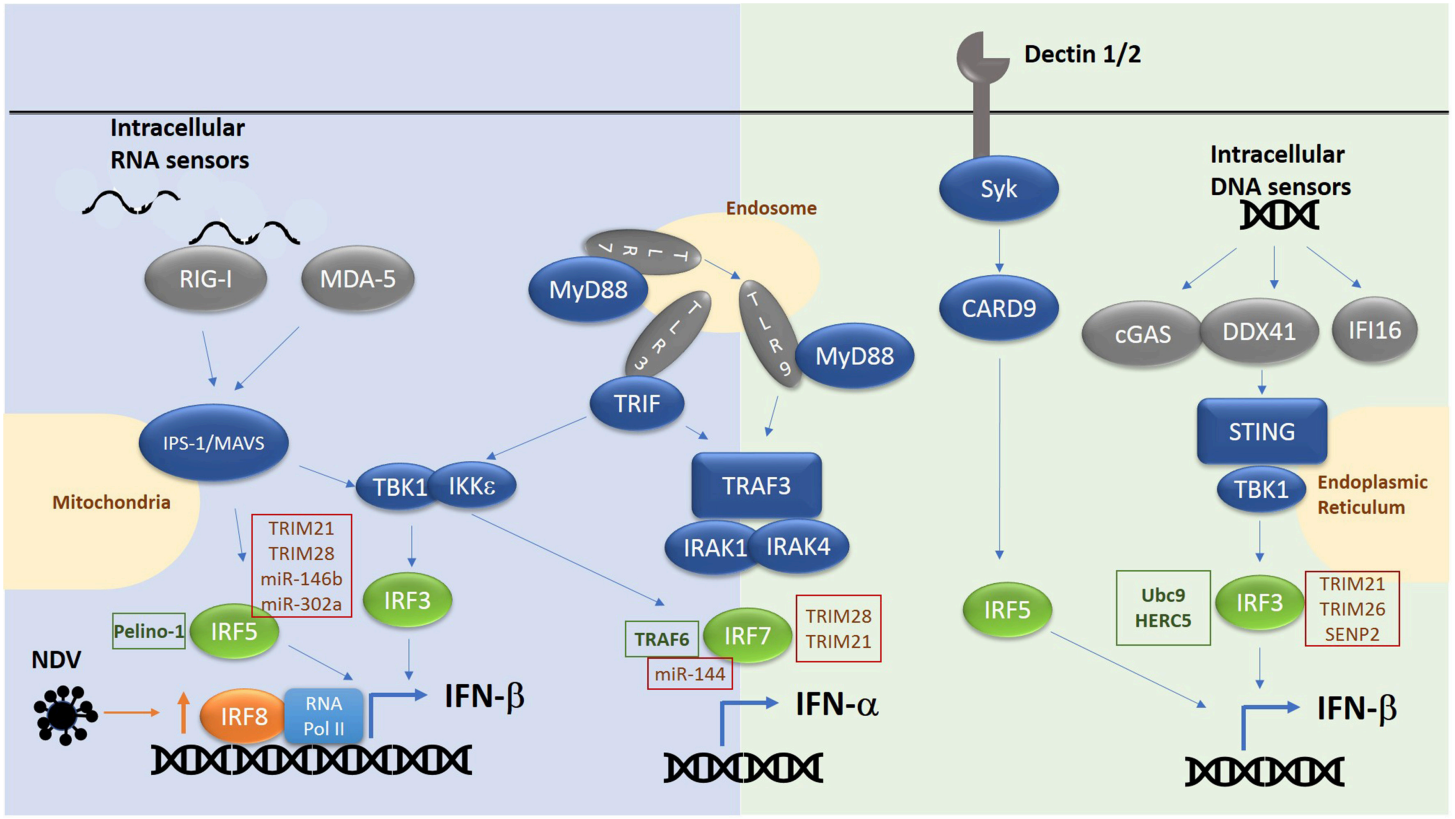
Overview of RNA/DNA sensing pathways. E3 ligases and microRNAs regulating IRF family members are highlighted in text boxes. Green text box for positive regulators and red for negative regulators.

IRF3, IRF5, IRF7, and IRF8 have been shown to be positive regulators of type I interferon gene induction downstream of pattern recognition receptors [Figure 1, reviewed in (12)]. Whilst IRF1 was the first IRF to be identified as an inducer of type I IFNs (13, 14), subsequent analyses in *Irf*^−/−^ MEFs suggested IRF1 was non-essential for induction of IFNs in response to cytosolic viruses (15). IRF3 and IRF7, the two family-members with greatest structural homology, are now known to be the principal mediators of IFN induction, acting downstream of cytosolic RNA and DNA receptors and the TLRs (TLR3, TLR4, TLR7, and TLR9) (9). IRF3 is ubiquitously expressed, whereas IRF7 is expressed only at very low levels, except in plasmacytoid DCs (pDCs) where it is relatively abundant (16). However, IRF7 expression is induced by type I IFNs, resulting in a feedforward loop that maximally drives type IFN expression (17). IRF3 is activated by phosphorylation (by kinases TBK1 and IKKε), promoting dimerization, nuclear translocation, association with the co-activator CREB-binding protein (CBP) and binding to canonical interferon response element sequence (IRES) in the promoter of IFN-β and IFN-α (18–21). Interestingly, a two-step phosphorylation of IRF3 has been proposed which involves TBK1 phosphorylation at site II (threonine 405 or serine 406) to relieve an autoinhibitory loop and promoting interaction with its co-factor Creb binding protein (CBP) and facilitating phosphorylation and full activation at site I (serine 385/386) (22–25). Activation of IRF3 occurs at intracellular vesicles via assembly of adaptor complexes, which then recruit in TBK1 and IKKε. TLR3 and TLR4 both use the adaptor protein TRIF to recruit in TBK1 to endosomes and phagosomes respectively, whereas RIG-I/MDA5 recruit the adaptor protein IPS-1 to recruit and activate TBK1 at the mitochondrial membrane. The growing number of cytosolic DNA-detecting PRRs (c-GAS, DDX41, IFI16) utilize the adaptor protein STING, found in the ER membrane, which once activated, translocates to the Golgi membrane to recruit and activate TBK1 (26). IRF3 can also directly induce the expression of cytokines in addition to type I IFNs, including CXCL10, RANTES, ISG56, IL-12p35, IL-23, and IL-15, whilst inhibiting IL-12β and TGF-β (27–33). However, it is currently unknown whether IRF3 activation can modulate the expression of these additional cytokines in all cells and downstream of all PRRs.

In a similar manner IRF7 is activated by TBK1/IKKε downstream of cytosolic RNA/DNA sensors and TRIF dependent pathways. Here IRF7 can either homodimerize or heterodimerize with IRF3 to induce IFN-α/β expression (34). Previously it was thought that, IRF7 was not required for IFN-β expression in the early phase of a response due to its low basal level in resting cells, and that IRF3 in complex with CBP alone was required. However, consistent with a role for IRF7 as the master regulator of IFN responses (34), we now know from work in *Irf7*^−/−^ MEFs that IRF7 in complex with IRF3 and CBP is essential for both the early and late phase induction of IFNs in response to single stranded RNA viruses. In pDCs in which the TLR7/TLR9 pathway is predominantly active, phosphorylation and activation of IRF7 is independent of TBK1/IKKε and instead involves recruitment of MyD88, recruitment and activation of IRAK1/2/4 signaling complex, resulting in IKKα activation and phosphorylation of IRF7, thus driving IFN-α/β expression in response to ssRNA or DNA viruses (35).

Together with IRF3 and IRF7, IRF5 is another important member of the family involved in driving IFN production. Indeed, a risk haplotype of IRF5 is associated with SLE and results in enhanced production of type I IFN. IRF5 is expressed predominantly in B cell, monocytes, macrophages and pDCs. Activation of IRF5 involves phosphorylation by IKKβ (36, 37) at conserved residues in the IAD domain. Similar to IRF3 and IRF7, this releases an autoinhibitory loop, promoting nuclear translocation and interaction with CBP. For example, mice lacking *Irf5* showed increased levels of type I IFN in their serum following infection with the RNA viruses vesicular stomatitis virus (VSV) or Newcastle disease virus (NDV) (38). This implicated the RIG-I like receptor signaling pathway in activating IRF5, which was confirmed by over-expression of MAVs inducing IRF5 activation and IFN-induction (36). In addition, bacterial sensing via nucleotide-binding oligomerization domain containing (NOD)2 has been shown to drive IRF5 phosphorylation (both via TBK1 and RIP2), leading to enhanced type I IFN expression (39, 40). In pDCs IRF5 is key to the induction of pro-inflammatory genes (IL-12, IL-6, TNF-α, and IL-23) downstream of TLR7/9-MyD88, featuring ubiquitination of IRF5 by TRAF6. Whereas, IRF7 is activated from late endosomes in response to TLR7/9 ligation to drive IFN expression, IRF5 is activated from early endosomes to drive inflammatory gene expression by binding MD88 directly, which in turn facilitates its ubiquitination and activation. Interestingly, IRF4 binds same region of MyD88 as IRF5 and negatively regulates MyD88 dependent signaling (41). IRF5 is also involved in driving IFN-β expression downstream of C type lectin receptors (CLRs) such as Dectin-1 and Dectin-2 which recognize the β-glucan cell wall of *C. albicans* (42). Such production requires Syk and Card9 in addition to IRF5 but is independent of other IRFs.

Thus, the co-ordinate activity of IRF3, 5, and 7 downstream of the various PRRs determines the extent of type I IFN induction and the pattern of cytokines induced. As to which IRF is activated in any given situation depends on both the initiating signal and the cell type involved. For example, in NDV-infected cells the IRF5/IRF7 heterodimer has an inhibitory effect on the *IFNA1* promoter, while IRF3 and IRF5 cooperatively activate this promoter (43, 44). In addition, overexpression of IRF5 or IRF7 results in expression of a different set of IFN-α subtypes, with IRF5-overexpressing cells driving mainly IFN-α8 expression, while IRF7-overexpressing cells produce mainly IFN-α1 (45). Thus, the potential exists that different levels of expression of IRF family members in different infection and disease settings will determine the level and subtype of type I IFN being produced. Indeed, given the central role for IRF3, 5, and 7 in regulating IFN expression, it is not surprising that they have been implicated in diseases such as systemic lupus erythematosus (SLE), which are driven in part by overexpression of type I IFNs. IRF5, for example, has a strong genetic association with disease (46), and a risk haplotype which results in enhanced IRF5 expression in SLE was found to correlate with enhanced levels of proinflammatory cytokines released from monocyte-derived cells from healthy individuals stimulated with NOD2 and TLRs ligands, thus indicating the presence of a correlation between *IRF5* genetic variants and IRF5-mediated transcriptional regulation of cytokine genes (47). Similarly, increased association of IRF3 with the promoter of IL-23 results in increased expression of this cytokine in SLE monocytes (33). A non-synonymous SNP in *IRF*7 is associated with enhanced IRF7 activity and is associated with SLE (48).

A role for IRF8 in stabilizing the basal transcription machinery at type I IFN promoters to enhance IFN expression in dendritic cells (DC) and monocytes has also been reported. Whilst principally known for its role in proinflammatory gene induction, IRF8 also reportedly takes part in a second phase of interferon induction in dendritic cells in response to Newcastle Disease virus (NDV) which triggers IFN induction via activation of RIG-I dependent pathways (49). The role of IRF8 in DC-induced IFN-β requires upregulation of IRF8 expression in a feed forward loop which then works via prolonging the recruitment of the basal transcription machinery to the promoters of IFN genes in dendritic cells. This mechanism is also at play in monocytes (50). Indeed, original investigations into a possible role for IRF8 in DC function supports a role for IRF8 in mediating the development of IFN-inducing DC subsets (51–53). However, it should be noted that the role of RIG-I in IRF8-mediated IFN induction in DCs may be indirect, driving the expression of IRF8 for example rather than directly activating this transcription factor.

### Signaling Downstream of IFN Alpha Receptor (IFNAR)

**Figure 2:**
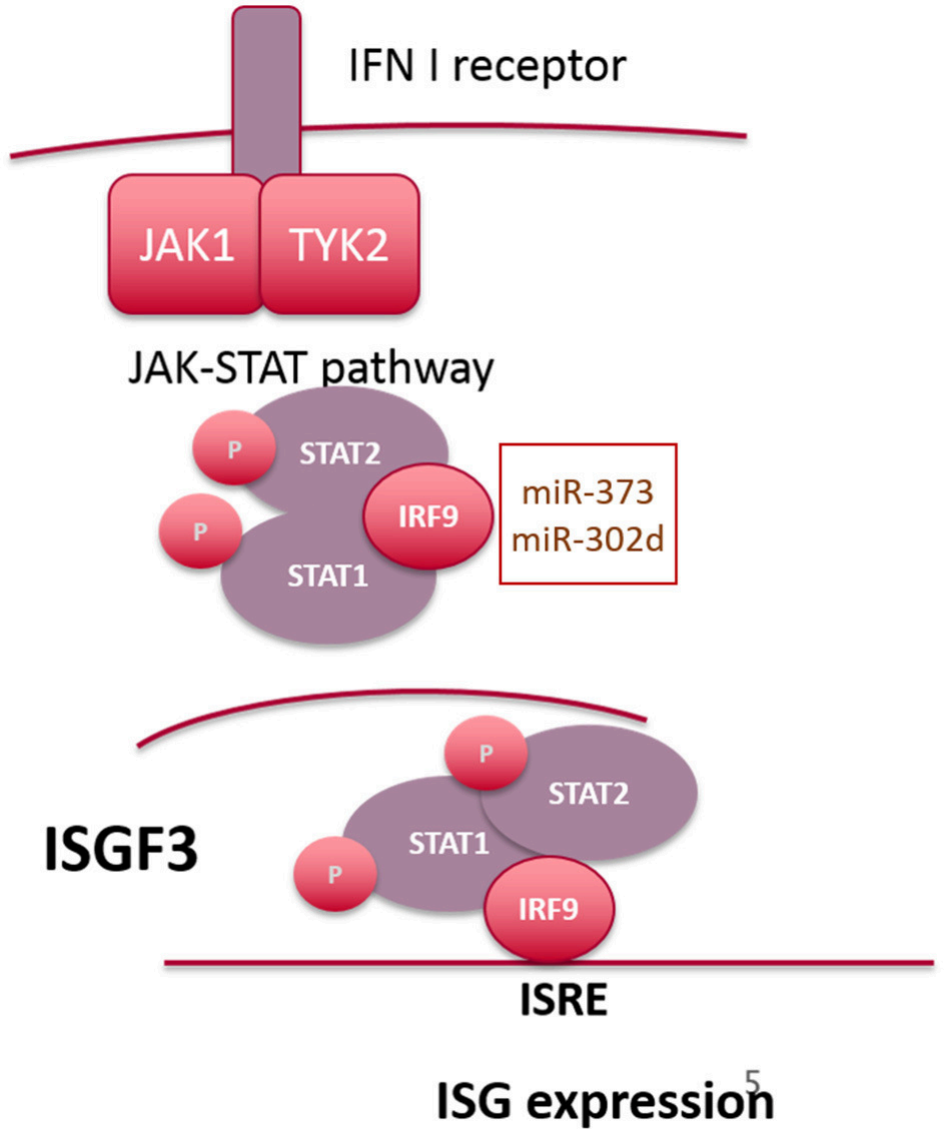
Overview of signaling downstream of the IFN-alpha receptor. microRNAs targeting IRF9 are highlighted in the text box.

Canonical type I IFN signaling occurs following binding of IFN to the ubiquitously expressed type I IFN receptor (IFNAR), comprising two transmembrane proteins, IFNAR1 and IFNAR2 (54). This results in activation of two cytoplasmic kinases JAK1 and TYK2, which subsequently phosphorylate the associated transcription factors STAT1 and STAT2 (55). Once phosphorylated STAT1 and STAT2 dimerize and interact with IRF9 to form the transcriptionally active complex, ISGF3, which binds to IFN-stimulated response elements (ISRE) in the promoter region of IFN-inducible genes (56, 57). In the ISGF3 complex, DNA binding activity is facilitated by IRF9, with STAT1 providing additional DNA contacts, thus stabilizing the complex (58). STAT2 provides a transactivation domain to enhance RNA pol II dependent gene expression but is unable to bind directly to DNA (Figure 2). In addition to ISGF3-dependent gene expression, STAT1 homodimers facilitate transcriptional responses to IFN-γ (and type I IFNs to a lesser extent) by binding to the IFN-γ activated site (GAS) DNA element. A type I IFN gene signature in the peripheral blood of SLE patients has been described which correlates with increased disease activity (59–61). This may result from enhanced levels of IFN-α or -β, or from constitutive activity of the JAK-STAT pathway, downstream of the IFNAR complex. For example, the JAK-STAT pathway has been shown to be activated in SLE patients (skin and kidney, specifically) (62–64) and in murine models (65, 66), with elevated levels of STAT1 protein detected both in monocytes and skin lesions from SLE patients. With respect to ISGF3, in a mouse model of pristane-inducible IFN-driven lupus, both IRF9 and STAT1 were shown to be required for autoantibody production and development of kidney disease (67).

Interestingly, the long-held paradigm that IFNα-driven tyrosine phosphorylation of both STAT1 and STAT2 is a prerequisite for interaction with IRF9 (68) has recently been challenged [reviewed in (69)]. For example, STAT2 is also capable of STAT1–independent ISRE-dependent gene expression, forming homodimers that interact with IRF9 following phosphorylation in response to IFN-α (70). However, Cheon et al. have recently demonstrated that increased expression of STAT1 and STAT2 as a result of constitutive low level IFN-β expression gives rise to a novel transcriptional complex composed of unphosphorylated STAT1 and STAT2 complexed to IRF9 (71), which drives a subset of anti-viral genes that overlap directly with the most highly expressed ISGs thus far identified in SLE patients. Although many of these studies were conducted in non-immune cells, they reveal the complexity of gene expression patterns downstream of the IFNAR receptor complex and highlight the possibility that overexpression of STAT1, STAT2, or IRF9 can have a profound effect on ISG expression and potentially allow ISG expression independent of signaling through IFNAR.

### Role for IRFs in IFN-Driven Autoimmune Disease

The role of IRFs in infection, protective immunity and primary immunodeficiencies has been reviewed extensively elsewhere in this focused issue (72). Given the role of IRF proteins in regulating both the production and downstream signaling of type I (and type II) interferons, it is hardly surprising that they have been both genetically and biochemically shown to be important mediators of IFN driven autoimmunity (4, 73, 74). Systemic lupus erythematosus (SLE) is amongst the best characterized for the involvement of IFNs in disease pathology. For example, in SLE, elevated IFN-α is observed in over 50% of patients and correlates with disease severity, flare and tissue involvement (specifically skin, kidney, and central nervous system). In recent years a type I IFN gene signature in the peripheral blood of SLE patients has been described which correlates with increased disease activity (59–61). More recently, Rheumatoid Arthritis (RA) patients have been found to have a type I IFN signature which correlates with autoantibody production (75), indicating that type I IFNs play an important role in driving a subset of RA (75). The various effects of type I IFNs on both the innate and the adaptive immune system contribute to the breaking of immune tolerance to self, overactivation of myeloid cells, B and T lymphocytes and differentiation or polarization of myeloid cells (monocytes and neutrophils) and T cells to more pathogenic sub-types. With respect for a role for IRFs in mediating these effects, genetic association studies have identified IRF5 and IRF7 as being risk factors for developing SLE (76–80). IRF5, like IRF7, is an IFN-inducible gene and is found to be significantly upregulated in PBMCs from SLE patients compared to healthy controls. IRF5 was found to be constitutively activated in monocytes from SLE patients resulting in enhanced levels of IL-6, TNF-α, and IFN-α (81, 82). IRF5 has been shown to be critical for the development of SLE in MRL-LPR mice (80), with alteration in function or expression of IRF5 affecting both myeloid cells and B cells in SLE-like models (79, 83). The role for IRF7 has been suggested not only for is critical role in regulating IFN-a production by pDCs, but also genetic association studies showing certain SNPs in IRF7 to confer enhanced risk for developing SLE. Functionally these genetic variants were found to be associated with increased serum IFN-α in SLE patients with autoantibodies against DNA and the Smith autoantigen (84). Interestingly, IRF3 has also been shown to be associated with enhanced IFN-α levels in SLE patients, the study also identifying a novel genetic association in a Mexican cohort of SLE patients, suggesting that IRF3 may play an important but as yet underappreciated role in driving IFN expression in SLE (85). IRF3 is also strongly associated with RA—elevated levels of phosphorylated IRF3 have been identified in the synovial tissue of RA patients and IRF3 has also been strongly associated with ISG expression in RA (86, 87). Regarding a role for other IRFs in IFN-driven disease, we recently demonstrated that IRF9 expression is enhanced in SLE monocytes and positively correlates with ISG expression (88), indicating that perturbations of IRF9 levels may alter functional activity of the ISGF3 complex and potentially contribute to disease activity. The ability of IRFs to regulate IFN production and downstream signaling thus makes them important potential targets for therapeutic intervention—highlighting the importance of understanding how their activity is controlled in molecular detail. One aspect that is rarely considered in IRF biology is the effect that conventional treatments for autoimmune diseases via their ability to alter IFN expression may also affect the expression of IRFs in patients, given the fact that IRF3, 5 7, and 9 are all IFN-regulated genes. For example, glucocorticoids, the mainstay treatment for autoimmune and inflammatory disorders, inhibit the expression of IFN stimulated genes. They therefore not only alter the expression of IFN-regulated IRFs but can directly impact their activity by targeting an interaction between the glucocorticoid-sensitive coactivator GRIP1/NCOA2 and IRF family members—IRF9 and IRF3 specifically (89, 90). Thus, in IFN driven diseases glucocorticoid treatment would be expected to reduce the expression and activity of the IFN signature as has been shown for SLE (91) and RA (92). Another mainstay for treating IFN-driven diseases (particularly SLE) also has a direct effect on the expression of IFNs and can therefore affect IRF levels. These are the anti-malarial 4-aminoquinoline drugs chloroquine and hydroxychloroquine which accumulate in the endolysosomal compartment of cells and inhibit signaling of endosomal TLRs such as TLR3, 7, and 9 and hence IFN-induction. In SLE, patients on chloroquine/Plaquenil show a reduction in IFN levels and would therefore be expected to show corresponding changes in IFN-regulated IRF expression (93). Interestingly, chloroquine is implicated in directly regulating IRF3 activity via increased expression of the deubiquitinating enzyme USP25, which enhances IRF3 nuclear translocation and results in increased LPS-induced IFN-β expression (94). This raises the possibility that chloroquine can directly or indirectly affect the activity and expression of IRF proteins in SLE or other IFN-driven diseases.

Several anti-IFN therapies have been clinically evaluated in SLE in recent years with varying degrees of success. Sifalimumab improved disease in patients with moderate to severe active disease, reducing the level of IFN stimulated genes (ISGs) in patients with initially high ISG scores, whereas the effects of Rontalizumab were greatest in patients with low to moderate levels of ISGs (95, 96). Anifrolumab, a blocking antibody against the IFN receptor (as opposed to targeting IFN-α isoforms), has reportedly better efficacy, although responses are far from complete (97). Another contribution IFN-driven disease that cannot be discounted in the potential role of intracellular RNA/DNA receptors in regulating type I IFN production. The recent identification that mutations in *STING* or *TREX1* (which both work to regulate IFN-β production) drive monogenic forms of IFN-driven disease (interferonopathies) have suggested that dysregulation of these pathways may contribute to interferon driven diseases such as SLE or Sjogren's syndrome (98). Indeed, DNA released from stressed mitochondria in SLE neutrophils has been shown to drive IFN responses via the cGAS-STING pathway (99–101). More recently the cGAS-STING pathway has been shown to contribute to ISG regulation, independent of type I signaling through the IFNAR complex, indicating that other mechanisms may be at play in driving ISG expression in cells (102). Whether cGAS-STING activation of IRF3/IRF5 drives ISG expression directly in this scenario, or whether it drives expression of type III IFNs (IL-28A, IL-28B, and IL-29) which can also drive expression of ISGs (103), remains to be fully explored. These studies highlight the need to understand these pathways in molecular detail and underscore the complexity of targeting the IFN system therapeutically.

## Immune Cell Development and Differentiation

**Figure 3:**
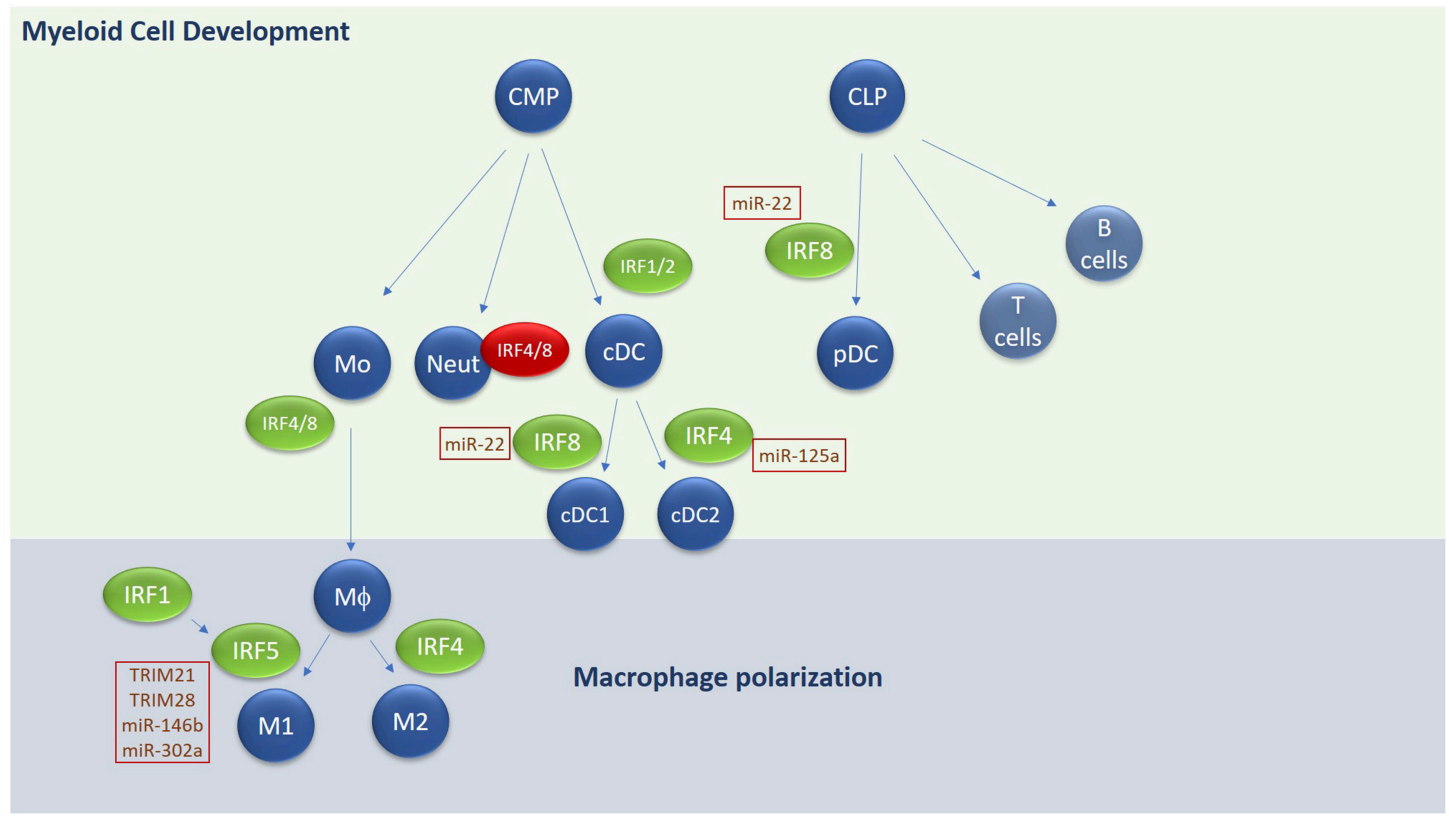
Overview of IRF involvement in myeloid cell development and macrophage differentiation. E3 ligases and microRNAs regulating IRF family members are highlighted in text boxes. Green text box for positive regulators and red for negative regulators. CMP, common myeloid progenitor; CLP, common lymphoid progenitor; Mo, Monocyte; Neut, Neutrophil; DC, Dendritic cell; MΦ, macrophage.

In addition to regulating IFN production IRFs have important roles in regulating immune cell development and differentiation (Figure 3). Whilst IRFs have been shown to regulate both lymphoid and myeloid cell development and differentiation, possibly their most influential role is observed in regulating dendritic cell (DC) subset development and macrophage differentiation/polarization, with obvious consequences for inflammatory outcomes.

### Myeloid Cell Development

Hematopoietic stem cells give rise to both the myeloid and lymphoid arms of hematopoietic lineage. Myeloid cells derive primarily from the Common Myeloid Progenitor (CMP) whereas the lymphoid arm derive from the Common Lymphoid Progenitor (CLP). The CMP can give rise to all types of myeloid cells, including monocytes, neutrophils and most types of dendritic cells (DCs). A unique subset of DCs, termed plasmacytoid DCs derive from CLP. IRFs play an integral role in both DC and monocyte development. DCs are essential for antigen presentation and act as the bridge between innate and adaptive immune responses. They comprise four main subsets of DCs—conventional DCs (cDCs), plasmacytoid DCs (pDCs), monocyte-derived DCs, and Langerhans cells. Conventional DCs in mice are further sub-grouped into cDC1 and cDC2 subsets with different markers for human and murine counterparts (104).

Each DC subset develops under the control of differential expression of IRF4 and IRF8 in collaboration with transcription factors such as PU.1, ID2, and KLF4 (105–108). For conventional DCs, IRF8 regulates cDC1 subset development in mouse and humans, characterized by expression of CD8 or CD103 in mice or CD141 in humans and by the expression of IL-12 following TLR engagement. IRF4 on the other hand regulates cDC2 subsets, which express high levels of CD11b and CD172 in both mouse and humans and are highly efficient at inducing CD4^+^ T cell effector function and expansion. High expression of IRF8 in combination with E2-2 and Bcl11A are required for development of pDCs, which secrete high amounts of type I IFN in response to stimulation. IRF1 and IRF2 also appear to be important in regulating DC subset development—*Irf* ^−/−^ mice show a loss of splenic and epidermal DCs (due to augmented type I IFN signaling) (109, 110) whereas *Irf1*^−/−^ exhibit an increase in pDCs and a decrease in CD8^+^ DCs in mice, along with an increase of IL-10 and TGF-β (111). In addition to regulating DC differentiation, IRF8 also promotes the commitment of myeloid progenitors to the monocyte/macrophage lineage, whilst inhibiting development of neutrophils (112). *Irf8*^−/−^ mice lack bone marrow resident macrophages, in addition to CD8^+^ DCs and pDCs in lymphoid organs (53, 113, 114). IRF4 has also been shown to promote macrophage differentiation and impair granulocyte formation, but its role in these events is secondary to IRF8 (115). A recent role for IRF4 in negatively regulating myeloid-derived suppressor cell (MDSC) development and immunosuppressive function in tumors has recently been described (116), indicating the importance of understanding IRF-dependent regulation of myeloid cell development and function for disease.

### M1/M2 Macrophage Polarization

Like DCs, macrophages play an important role in sensing pathogens, initiating innate immunity, and cross-talking with the adaptive immune system to generate an appropriate immune response. Like DCs and T cells, subsets of macrophages with differing functions have been identified [reviewed extensively in (117, 118)]. Broadly speaking they can be divided into inflammatory M1 macrophages and anti-inflammatory or resolving M2 macrophages. M2 macrophages can be further subdivided into M2a-M2d subsets. Stimuli such as GM-CSF, LPS, and IFN-γ are potent drivers of M1 polarization for example, whereas fungal products, immune complexes, M-CSF and IL-4, IL-13, IL-10, and TGF-β all promote M2 macrophages. M1 macrophages are characterized as secreting high levels of TNF-α, IFN-γ, IL-12, and IL-23, promoting strong microbiocidal functions and production of reactive nitrogen and oxygen species and promotion of Th1/Th17 responses. In contrast M2 macrophages regulate parasitic infections, promote tissue remodeling and repair and secrete immunosuppressive cytokines IL-10 and TGF-β. Regarding the different M2 subsets, M2a subtype is driven by IL-4, IL-13, and fungal and helminth infections. M2b is driven by immune complexes, IL-1/IL-18 and LPS, whilst M2c is elicited by IL-10, TGF-β and glucocorticoids. Functionally, M2a and c secrete IL-10 and TGF-β and are generally immunosuppressive, whereas M2b secrete IL-1, IL-12, and IL-10 and are implicated in inflammatory diseases such as SLE. M2d macrophages have only been identified in mice thus far and are induced from M1 macrophages following exposure to ATP (119, 120). Phenotypically they play a role in tissue remodeling and repair and have been shown to be associated with angiogenesis through secretion of VEGF (121).

Regarding IRF involvement in M1/M2 differentiation, IRF4 is strongly associated with M2 polarization, interacting with other transcription factors and chromatin remodelers to drive M2a or M2c subsets (122). The histone demethylase Jumonji domain containing-3 (Jmjd3) is involved in depressing M2-associated genes by reversing epigenetic modifications and has been shown by Satoh *et al* to work in concert with IRF4 to induce M2 polarization (123). Both IRF4 and Jmjd3 induce expression of M2-specific genes, arginase 1, FIZZ1, Ym1, and mannose receptor (MR) in response to IL-4 stimulation. Both Jmjd3 and IRF4 expression is driven by IL-4 in macrophages, and they in turn reciprocally regulate expression of each other (123, 124). Thus, IRF4 and Jmjd3 regulate M2a polarization downstream of IL-4 and IL-13. IRF4 also antagonizes IRF5 binding to MyD88 and in this way promotes M2 over M1 differentiation (125). Whether IRF4 is required for M2b, M2c, or M2d polarization is currently unknown.

IRF5 is the key transcription factor regulating M1 polarization (126, 127). Various inflammatory stimuli such as GM-CSF, LPS, and IFN-γ can upregulate the expression of IRF5. Enhanced expression of IRF5 in M1 macrophages is required to drive transcription of M1 markers such as IL-12, TNF-α, and IFN-γ and repress IL-10 (128). IRF5 has also been shown to regulate IL-23 secretion from macrophages, thus triggering the differentiation of Th17 cells (126). Thus, by influencing macrophage polarization toward an M1 phenotype, IRF5 plays an important role in regulating downstream adaptive immune responses and T helper cell differentiation toward a Th1 or Th17 phenotype. IRF1 seems to facilitate M1 polarization in general—priming expression of inflammatory genes associated with an M1 phenotype, such as IL-12p35 and IL-12p40 and synergizing with IRF8 to drive IL-12 production. IRF1 can also directly co-operate with IRF5 in order to drive M1 polarization in response to IFN-γ (125) and IRF1 and IFN-β work together to enhance IRF5 expression and as a consequence, M1 polarization in U937 cells. Thus, IRF1 promotes M1 polarization through its ability to enhance IRF5 levels and activity. The ability of IRF4 to compete with IRF5 for MyD88 binding and hence activation of downstream signals, suggests that relative levels of IRF4 and IRF5 in macrophages are important determinants of whether cells will polarize toward M1 or M2 phenotype.

## Post-Translational Regulation of IRFs—Ubiquitination and Non-coding microRNA

Given the critical function of IRF family members in regulating IFN production and downstream signaling, and their role in regulating immune cell differentiation, means to regulate their activity are critical to preventing overstimulation of pathways and cells and consequent autoimmune disease. We will discuss two mechanisms to negatively regulate IRF family members—the post-translational modification of IRFs by ubiquitin and ubiquitin-like proteins and the epigenetic mechanism of microRNA (miR) targeting.

### Ubiquitination

Ubiquitination, like phosphorylation, is a reversible process regulated by E3 ligases that add ubiquitin chains to targets and de-ubiquitinases that remove these chains [reviewed in (129, 130)]. Ubiquitin itself is a small, ubiquitously expressed, 76 amino acid (8.6 kDa) protein that is conjugated to an internal lysine of a target via the formation of an isopeptide bond between its C terminal glycine reside and the ε-amino residue of the lysine on the target protein. Ubiquitin chains are then formed on this initiating ubiquitin and the internal lysine targeted for polyubiquitination determines function—for example Lysine 48 (K48) linked chains target the protein for degradation, whereas K27 and K63 linked chains alter the activity of the protein target. Again, like phosphorylation, ubiquitination is a rapid method for activating or deactivating pathways. Indeed, signaling downstream of the PRRs is widely regulated by ubiquitination, both in order to activate signaling and to turn it off pathways once the response is deemed sufficient (131, 132). For example, the adaptor protein STING is regulated by multiple E3 ligases such as TRIM56, TRIM32 and AMFR, each activated by specific pathways in order to confer a specific outcome—i.e., STING activation, inactivation or relocalization. TRIM56 and TRIM32 catalyze K63-linked polyubiquitination of STING, driving dimerization and promoting its ability to interact with TBK1 and drive IFN-β expression (133, 134). K48-linked ubiquitination of STING by RNF5 and TRIM30a has also been reported, resulting in proteasomal degradation of STING and subsequent downregulation of cytosolic DNA-mediated signaling and IFN production (135, 136). AMFR on the other hand, in complex with INSIG1, catalyzes K27-linked polyubiquitination of STING, which acts as a platform to recruit in TBK1 and facilitating translocation to perinucleosomes and antiviral gene expression (137). Recently, ubiquitination of STING on K224 by the E3 ligase MUL1 has recently been shown to regulate its trafficking from the endoplasmic reticulum (ER) to the Golgi (138). In addition to ubiquitin, SUMO (small ubiquitin-like modifier) can also be covalently linked to lysine residues in target proteins, acting to regulate localization, protein-protein interactions, and activity of target proteins, a process known as SUMOylation. Indeed, TRIM38 has also been shown to regulate SUMOylation of STING during early responses to DNA virus, to promote its stability and enhance its activity (139). Thus, ubiquitination of proteins or addition of ubiquitin-like modifiers such SUMO is a highly dynamic, versatile, and effective means of regulating protein function and levels in cells.

The activity of IRF proteins is tightly controlled through both ubiquitination and SUMOylation. In general, ubiquitination and phosphorylation of IRFs are integrally linked, with one modification often being a pre-requisite for the other to take place (140). For example, ubiquitination of IRF7 by TRAF6 at lysine 444, 446, and 452 is required prior to TBK1/IKKε driven phosphorylation at serine 477 and 479 (141). The juxtaposition of both the ubiquitination site and phospho-acceptor site on IRF7 and other IRFs suggests that such these post-translational modifications work sequentially to recruit in all the players necessary for activation. And similar to STING, it appears that competing ubiquitin or ubiquitin-like modifications work to fine-tune and regulate IRF protein stability and function. For example, both IRF3 and IRF7 are negatively regulated by SUMOylation following viral infection in order to turn off and limit responses (142). TRIM28 is the E3 ligase that regulates IRF7 SUMOylation at K444 and K446 (143).

Regulation of IRF3 activity by ubiquitination or other ubiquitin like modifiers such as SUMO or ISG15, is highly complex, and most likely is highly dependent on context and cell type. IRF3 stability is regulated by K48-linked ubiquitination by TRIM21 promoting proteasomal degradation post TLR-stimulation in order to turn off and limit responses (144). Indeed, TRIM21 deficient mice develop SLE-like symptoms, accompanied by enhanced IFN levels, accompanied by sustained IRF3 levels post TLR-activation (145). TRIM21 also plays a role in autophagy and has been shown to interact with the p62 sequestersome protein, thus facilitating removal of IRF3 by targeted autophagy (146–148). In contrast, TRIM21 ubiquitination of IRF3 has also been shown to stabilize IRF3 activity via disrupting an interaction between IRF3 and Pin1, a protein that promotes IRF3 degradation (148–150). Both published and unpublished results from our group indicate that TRIM21-mediated regulation of IRF3 is complex and that it may in fact act to stabilize IRF3 in resting cells (as evidenced by decreased basal levels of IRF3 in TRIM21-deficient BMDMs) but then become activated, potentially by phosphorylation (151), to promote ubiquitination and proteolysis of IRF3 in order to limit and turn off anti-viral responses. TRIM21 also regulates IRF7 stability downstream of viral TLRs in order to limit antiviral responses (152). Like TRIM21, the E3 ligase RAUL adds K48-linked ubiquitin chains to both IRF3 and IRF7 and ultimately acts as a brake on the system in response to viral infection (153).

Similar to IRF7, IRF3 is also regulated by other ubiquitin-like modifiers: addition of SUMO and another modifier interferon stimulated gene 15 (ISG15) to on the N terminal DBD works to sustain IRF3 levels by protecting these sites from ubiquitination. Ubc9 for example SUMOylates IRF3 (142) whilst SENP2 is a deSUMOylating enzyme that removes SUMO for IRF3, presumably then allowing TRIM26 to ubiquitinate these residues with K48-linked chains, promoting IRF3 degradation (154, 155). ISGylation of IRF3 by HERC5 inhibits the interaction between IRF3 and PIN1, thus preventing Pin1-dependent IRF3 degradation (156). Thus, competing ubiquitin-like modifications on IRF3 work to either stabilize or degrade IRF3.

IRF5 stability is also regulated by ubiquitination. K63-linked ubiquitination of IRF5 by Pelino-1 for example positively regulates M1 polarization downstream of TLR4/IFN-γ. This study also linked the Pellino-1-IRF5 axis to regulation of glucose intolerance in obesity, with BMDMs from mice lacking Pellino-1 showing improved glucose intolerance when fed a high-fat diet (157). Work from our own lab has shown that TRIM21 differentially ubiquitinates different isoforms of IRF5, with IRF5-V1 and V-5 targeted or degradation by TRIM21 whereas IRF5-V2 and IRF5-V3 (IRF5-V2 linked to susceptibility to SLE) are resistant to TRIM21-mediated degradation, with obvious implications for downstream activity (158). TRIM28, a SUMO E3 ligase, is an additional negative regulator of IRF5 activity, promoting epigenetic modifications of IRF5-dependent genes (159).

Interestingly, ubiquitination of IRF1 is linked with stability and seems to be required for IL-1-induced expression of the chemokines CXCL10 and CCL5, thus promoting inflammatory cell recruitment (160). The E3 ligase responsible is the apoptosis inhibitor cIAP2, whose activity is enhanced by the sphigosphine-1-phosphate, catalyzing the addition of K63-linked chains onto IRF1. Recently Src family kinases have been shown to positively regulate K63-linked ubiquitination and accumulation of IRF1 in response to TLR7/8 signaling in monocytes and B cells (161).

As to whether other IRF proteins that are involved in regulating IFN production or downstream signaling pathways are regulated by ubiquitin-like post-translational modification remains to be determined. Given the fact that type I IFNs themselves rapidly induce expression of both E3 ligases [particularly the TRIM family (162)] that target IRFs, it is hardly surprising that many of these mechanisms are being considered as targets for therapeutic intervention in diseases driven by interferons such as SLE.

### microRNAs Targeting IRF proteins

microRNAs (miRs) are important regulators of gene expression in a whole host of cellular processes and immune responses (163, 164). They are an evolutionarily conserved family of small (~22 nucleotides long) non-coding RNAs that function to bind the 3′ UTR of mRNA targets and thus regulate gene expression. Like coding RNA, non-coding RNAs such as microRNA can be either constitutively expressed or inducible—and the inducibility of these small epigenetic modifiers allows cells to exquisitely regulate and control various pathways—including those regulated by IRF proteins. Binding can trigger degradation of the target mRNA (as occurs in the majority of cases), prevent translation, or in rarer cases, stabilize the mRNA leading to positive regulation. The biogenesis and functions of microRNA have been reviewed extensively elsewhere (165–168). The focus here will be to review the role microRNAs play in regulating the levels of IRF protein members and how this contributes to both homeostasis and to disease.

There is a body of evidence to support a role for miRs in the regulation of pathways producing type I interferons and those downstream of the IFN receptor complex. For example, miRs have been implicated at all levels of TLR signaling, including manipulation of TLR levels themselves (169, 170). Downstream of the TLRs, miR-146 has been shown to target a number of signaling molecules, including IRAK1 and TRAF6 (171–173). The ability of a single miR to target multiple players on a particular pathway is a unique feature of these epigenetic regulators and suggests that they have evolved to regulate pathways and processes in the cell rather than individual players. Regarding the IRFs that regulate IFN-α and -β production, both IRF5 and IRF7 have been shown to be targeted by specific miRs. miR-302a for example is induced by influenza A and targets IRF5 directly, in order to control and limit IFN production (174). Regarding regulating IRF5 to influence M1/M2 transition, IL-10 induces miR-146b, which in turn directly targets IRF5 to promote M2 differentiation (175). microRNAs that target IRF7 on the other hand have been linked to its role in regulating oncogenesis and apoptosis rather than IFN induction *per se*—for example in breast cancer cells, miR-762 targets IRF7, inhibiting proliferation and invasion in a matrigel assay (176). In a separate study, miR-541 was shown to promote vascular smooth muscle cell proliferation by targeting IRF7 and thus inhibiting apoptosis (177). Regarding how microRNAs might target IRF7 in order to regulate IFN production, miR-144 was shown to target the TRAF6-IRF7 axis, targeting TRAF6 in order to attenuate attenuating the host response to influenza virus, indicating that mechanisms to regulate IRF7 activity by microRNAs exist whether direct or indirect (178).

To date however, no miR has been uncovered that specifically targets IRF3—instead many have been identified that regulate upstream adaptor proteins and hence the activity of IRF3. For example, miR-3570 targets the adaptor protein IPS-1/MAVs in order to shut-off RIG-I dependent signaling. miR-576-3p was shown to be induced in response to RNA and DNA viruses via IRF3-depependent IFN-β production, in order to shut off and limit anti-viral responses. It achieves this by targeting STING, MAVS, and TRAF3, all 3 critical players in regulating IRF3 activity or facilitating type I IFN expression. Therefore, IRF3 drives a negative regulatory loop involving miR-576-3p (179, 180).

Regarding signaling downstream of the type I IFN receptor (IFNAR), IRF9 levels and activity are critical in mediating STAT1/STAT2 driven responses. A number of microRNAs have been published that target IRF9 directly. miR-373 for example is upregulated by Hepatitis C virus (HCV) and targets IRF9 and JAK1 in order to turn off and limit anti-viral defense mechanisms (181). Our own work has shown the IRF9 is also targeted directly by miR-302d. In this study we observed that miR-302d, an estrogen regulated microRNA, is decreased in SLE monocytes, resulting in enhanced expression of IRF9 (88). The level of expression of IRF9 positively correlated with levels of interferon stimulated gene (ISG) expression and also disease activity, indicating that disruption of the microRNA balance in cells may have important consequences for immune cell function, particularly in the context of autoimmune disease.

Regarding a role for microRNAs in targeting IRFs to influence myeloid cell development or differentiation, one would expect that targeting IRF4, IRF8, or IRF5 would directly influence these events. Indeed, as mentioned above, miR-302a targets IRF5 to influence M1/M2 levels in response to viral infection (174). miR-125a has recently been shown to regulate M1/M2 differentiation and inflammation, targeting negative regulators of inflammation such as A20 and promoting an M1 or pro-inflammatory phenotype (182, 183). A recent study showed that Notch-dependent upregulation of miR-125a in tumors inhibited tumor associated macrophage function and promoted M1 macrophages via its ability to regulate HIF1-a and IRF4 (184). Regarding regulating DC development or differentiation, IRF8 is the natural target as it positively regulates pDC over cDC. In this context, miR-22 directly targets IRF8 and was shown to be highly expressed in cDCs compared with pDCs and directly influence DC differentiation (185). Thus, understanding the role of microRNAs that target IRFs involved in myeloid cell function and development may have important relevance to disease pathology.

Given the numerous roles microRNAs play in fine tuning TLR and IFN responses, it is not surprising that the dysregulation of these molecules has been implicated in SLE. To date numerous examples of dysregulated SLE associated microRNAs have been identified (186–189). Best characterized in SLE are miR-146 and miR-125, which in addition to targeting IRF5 and IRF4, also upregulate IFN-α and RANTES, respectively, thus contributing to disease activity (190, 191). miR-125a, is downregulated in SLE, has been found to negatively correlate with levels of the chemokine RANTES, a major player in organ inflammation (192) and lupus nephritis (193). Investigations into the mechanism behind this revealed a role for miR-125a in negatively regulating Kruppel-like factor 13 (KLF13) expression, a transcription factor that binds and activates the RANTES promoter, thereby inducing its expression in T cells (194). Our own work has also confirmed *miR-125a* expression decreased in SLE monocytes and identified a novel target, IL-16, which regulates *CXCL10* expression in lung epithelial cells and helps drive lung inflammation in an autoimmune context (195). Given that monocyte and neutrophil subsets in SLE patients are key drivers of inflammation, understanding how microRNA changes in patients regulate IRF protein levels and hence contribute to myeloid cell development may be key in uncovering novel therapeutic targets.

## Future Perspectives

Numerous mechanisms exist to control the innate immune response and myeloid cell differentiation in order to prevent inflammatory and autoimmune disease. As IRF family members are critical in this respect, tight regulation of their levels and activity is one mechanism of maintaining tolerance to self-antigens such as self-nucleic acids. But in different diseases it appears individual IRFs have greater or lesser involvement [reviewed in (4)]. For example—IRF3 seems to be more important in synovial inflammation in RA and responsible for ISG induction, whereas its involvement in SLE does not seem to be as important. IRF5 may perhaps be more important in SLE. So rather than targeting a single IRF for all IFN-mediated diseases, we must first understand the complex interplay between the individual IRFs in specific diseases 9 and potentially sub-types of disease in order to understand how targeting individual family members will impact the immune response as a whole.

Regarding potential targeting strategies: Ubiquitination of IRFs is a rapid and versatile way to regulate both levels and activity of IRFs, whereas epigenetic targeting of IRFs by microRNAs can fine tune IRF expression levels. Both work in concert to tailor immune responses appropriately. However, many questions remain regarding the IRFs and how they are regulated as it pertains to IFN biology: for example—what role do IRFs play in IFNAR-independent induction of ISGs? Is it possible that different combinations of STATs and IRFs can replace the canonical ISGF3 transcriptional complex? What role does regulation of availability of IRFs by microRNA targeting play in this process? And finally, can we target E3 ligases to fine tune IRF function and levels? Answering these questions will undoubtedly contribute to our understanding regarding how IRFs contribute to the pathology of autoimmune diseases such as SLE, but its biggest impact will be in explaining the following: firstly how we can improve on current IFN-targeting strategies—i.e., will JAK inhibition provide enhanced efficacy compared with IFNAR targeting strategies? And secondly, potentially uncover additional new therapeutic targets—be they modulators of E3 ligase activity or RNA-targeting strategies. As central regulators of monocytes function and IFN biology, addressing these questions promises to have a big impact in IFN-driven autoimmune disease.

## Author Contributions

The author confirms being the sole contributor of this work and has approved it for publication.

### Conflict of Interest Statement

The author declares that the research was conducted in the absence of any commercial or financial relationships that could be construed as a potential conflict of interest.
